# Ultra-Processed Food Intake and Smoking Interact in Relation with Colorectal Adenomas

**DOI:** 10.3390/nu12113507

**Published:** 2020-11-14

**Authors:** Naomi Fliss-Isakov, Shira Zelber-Sagi, Dana Ivancovsky-Wajcman, Oren Shibolet, Revital Kariv

**Affiliations:** 1 Department of Gastroenterology, Tel Aviv Medical Center, Tel Aviv 6423906, Israel; zelbersagi@bezeqint.net (S.Z.-S.); orensh@tlvmc.gov.il (O.S.); revitalk@tlvmc.gov.il (R.K.); 2 Sackler Faculty of Medicine, Tel Aviv University, Tel Aviv 6997801, Israel; 3 School of Public Health, University of Haifa, Haifa 3498838, Israel; danaivanc@gmail.com

**Keywords:** ultra-processed food, diet, smoking, colorectal adenoma

## Abstract

Smoking and ultra-processed foods (UPFs), a substantial part of the western diet, have been suggested to have a potential carcinogenic effect, though epidemiologic data are lacking. We aimed to examine the association between high UPF intake and colorectal adenomas, and to test the interaction with smoking. In a case-control study among consecutive subjects undergoing colonoscopy in a tertiary center during 2010–2015, UPF intake and smoking were compared between cases with colorectal adenomas and controls. Within 652 participants (cases, *n* = 294 and controls, *n* = 358), high UPF intake (defined as percent of kcal from UPF above the study sample upper tertile) was positively associated with adenomas (Odds ratio (OR) = 1.75, 95% Confidence interval (CI) 1.14–2.68), advanced and proximal adenomas (OR = 2.17, 1.29–3.65 and OR = 2.38, 1.37–4.11) among the whole study sample; and with adenomas (OR = 3.54, 1.90–6.61), non-advanced adenomas (OR = 2.60, 1.20–5.63), advanced adenomas (OR = 4.76, 2.20–10.30), proximal adenomas (OR = 6.23, 2.67–14.52), and distal adenomas (OR = 2.49, 1.21–5.13) among smokers. Additionally, a dose-dependent association was observed between tertiles of UPF intake and adenomas only among smokers (*p* for trend < 0.001). A significant interaction between smoking and high UPF intake was detected (*p* for interaction = 0.004). High intake of UPFs is strongly and independently associated with colorectal adenomas, especially advanced and proximal adenoma, and interacts with smoking. Results highlight smokers as more susceptible to the negative health effects of UPF consumption on colorectal neoplasia.

## 1. Introduction

Colorectal cancer (CRC) is the world’s third most common cancer and the fourth most deadly cancer, with almost 900,000 deaths annually [1,2]. In addition to a genetic background, it has been shown that metabolic profile [3], lifestyle [4], dietary habits [5,6], and smoking [4,7] are strongly and independently associated with colorectal polyps, the direct precursors of CRC. Moreover, smoking and dietary factors have been reported to interact in their association with colorectal neoplasia [8,9,10].

A major component of the western diet is processed and ultra-processed food (UPF), contributing up to 79% of the mean daily calories [11]. According to the Food and Agriculture Organization of the United Nations, food processing includes physical, biological, and chemical techniques, used to prepare ready to eat, drink, or heat foods and beverages. UPF is energy dense; high in unhealthy types of fat, refined starches, sugar, salt, and artificial additives; as well as a poor source of protein, fiber, and micronutrients [12]. The most common UPFs are snacks, drinks, and ready-to-eat meals. Epidemiological studies indicate that high intake of UPFs is associated with increased risk for obesity [13], insulin resistance and metabolic syndrome (Mets) [14], dyslipidemia, hypertension, and cardiovascular disease [15]. Furthermore, UPF intake has been linked to overall cancer, particularly post-menopausal breast, prostate, and colorectal cancers [16,17], although that association has not been conclusively established. Therefore, we aimed to examine the association between UPF intake and several types of colorectal adenomas, and to test the interaction between UPF intake and smoking.

## 2. Materials and Methods

A case-control study, among consecutive subjects aged 40–70 years, undergoing colonoscopy at the Department of Gastroenterology and Hepatology at the Tel-Aviv Medical center (TLVMC) during 2010–2015 was conducted. We selected a population with minimal risk of genetic predisposition for colorectal neoplasia in order to assess the impact of environmental risk factors for CRC. Exclusion criteria for both cases and controls included: hereditary CRC syndromes (such as Lynch and Familial polyposis syndromes), a personal history of CRC, first-degree family history of CRC below the age of 70 years, inflammatory bowel disease, and celiac disease. Additionally excluded were those who had a history of solid malignancy, hyperthyroidism, or colectomy, as well as recent hospitalization or surgery, pregnancy, chronic liver disease, grade 4–5 chronic kidney disease, and type 2 diabetes. Cases with a personal history of colorectal polyps before the age of 40, or diagnosis of > 5 colorectal polyps (ever) were excluded, as well as controls with any past colonic polyps. Participants with excessive alcohol intake (≥30 g/day in men or ≥20 g/day in women), positive hepatitis serology, or an unreasonable food frequency questionnaire (FFQ) (total calorie intake of less than 500 or 800 kcal or more than 3500 or 4000 kcal for women and men, respectively) were also excluded.

The study protocol was approved by the Institutional Review Board of the TLVMC (Project identification code 0101-08-TLV, approved on 21.09.2009), and was therefore preformed in accordance with the ethics standards laid down in the 1964 Declaration of Helsinki and its later amendments. All participants provided informed consent prior to study enrollment.

### 2.1. Definition of Cases and Controls

Indication for colonoscopy was defined as screening (due to age), colonoscopy for alarming symptoms (such as rectal bleeding, unintentional weight loss etc.), or surveillance (due to a personal or family history of colorectal polyps or a family history of CRC past the age of 70 years).

Polyp histology was reviewed by a gastro-intestinal (GI) pathologist. Adenomas were classified as advanced or non-advanced according to the guidelines of the United States (US) Multi-Society Task Force on CRC, the American Cancer Society CRC Advisory Group, the US Multi-Society Task Force, and the American College of Radiology CRC Committee [18,19]. Advanced adenomas were defined as large adenomas (>10 mm), with features of high-grade dysplasia (HGD) or villous histology, multiple (≥3) non-advanced adenomas. Non-advanced adenomas were defined as adenomas <10 mm, without features of HGD or villous histology. Cases with more than one polyp were defined according to the polyp of highest neoplastic potential. Controls were patients with no colorectal polyps detected in their current or past colonoscopies.

Polyp location was defined as proximal adenomas (cecum, appendix, ascending colon, hepatic flexure, transverse colon, and splenic flexure) and distal adenomas (descending colon, sigmoid colon, and rectum) based on International Statistical Classification of Diseases 10th edition for CRC [20].

### 2.2. Data Collection

Within 2 months after undergoing a colonoscopy, all participants were requested to undergo a medical interview, anthropometric measurements, and blood tests and answer questionnaires on lifestyle and diet. Participants were face-to-face interviewed for their medical history, demographic characteristics, lifestyle, and dietary intake. Blood pressure, weight, height, and hip and waist circumference were measured using a uniform protocol. Body mass index (BMI) was calculated as weight (kilograms)/height^2^ (meters). Participants’ blood tests were obtained following a 12-h fast, and were analyzed at a single lab.

Smoking was defined as ever (past/present) smoking vs. never smoking.Mets was defined according to the American Heart Association (AHA) criteria [21] if patients were diagnosed with ≥3 of the following criteria: impaired fasting glucose (fasting glucose>100 mg/dl), hypertension (systolic blood pressure/diastolic blood pressure >130/85 mmHg and/or medication), low high-density-lipoprotein (HDL) (HDL <40/50 mg/dL among men and women, respectively), high triglycerides (triglycerides >150 mg/dL or medications), and abdominal obesity (waist circumference >88/102 cm among women and men, respectively). A healthy dietary index was defined according to the AHA healthy diet components [22,23].

### 2.3. Evaluation of UPF Intake

Evaluation of dietary intake was performed using a structured detailed semi-quantitative FFQ, assembled by the Food and Nutrition Administration, the Israeli Ministry of Health, that has been validated for the Israeli population [24], and comprised of 117 food items with specified serving sizes. All participants were asked to refer to the past year when filling in the FFQ, and were kept blinded to the study hypothesis.

The classification of the processing level of foods was determined based on the NOVA classification [25]. UPFs were classified as foods that are industrial formulations that are typically comprised of many ingredients, particularly substances not commonly found in natural food products, such as hydrolyzed protein, modified starch, hydrogenated or inter-esterified oils, and additives, such as colorants, flavoring, non-sugar sweeteners, emulsifiers, humectants, sequestrants, firming, bulking, de-foaming, anti-caking, and glazing agents [25] (Table S1**)**.

Total calories from UPF items were summed, and the proportional caloric intake of UPFs from total caloric intake, and separately for 6 food groups was calculated: (1) bread, pastries, and starch; (2) snacks; (3) beverages; (4) oils and spreads; (5) dairy; and (6) meat, poultry, and fish [13]. The proportional caloric intake of UPFs from total calories, and from each food group were categorized into tertiles according to the consumption of the study sample. High UPF intake was defined as percent of kcal from UPF above the study sample upper tertile (≥ 44.8% of total kcal).

### 2.4. Statistical Analysis

All statistical analyses were performed using SPSS version 25.0 for Windows (SPSS Inc., Chicago, IL, USA). Continuous variables are presented as means ± SD and nominal variables as proportions. Pearson Chi-Square test was used to test the association between nominal variables. The independent samples *t*-test was used to compare between cases and controls. One-way Anova was used to test the difference between three groups of proportional UPF intake. Multivariate logistic regression analysis was used to test the association between high UPF intake and colorectal adenomas, controlling for potential confounders as demographics and variables that distributed differently between cases and controls and may be related with dietary characteristics: age, gender, aspirin use, indication for colonoscopy, BMI, total kcal, and the Mets. The proportional caloric intake of UPFs from total calories, and from each food group was categorized as tertiles. For all UPF in the diet, the 2nd tertile (30.4–44.7% of total kcal) and the 3rd tertile (≥44.8% of total kcal) were compared to the 1st tertile (≤30.4% of total kcal). An interaction between high UPF intake and smoking status was assessed by the interaction term in binary logistic regression, adjusting for potential confounders, smoking status, and high UPF intake. *p* < 0.05 was considered statistically significant for all analyses.

## 3. Results

### 3.1. Characteristics of the Study Population and Comparison between Cases and Controls

We included 652 participants in this analysis, 294 cases with colorectal adenomas and 358 controls with no past/present polyps (mean age ± standard deviation (SD) 58.5 ± 6.6 years, 50.8% men, mean BMI 28.2 ± 5.4 kg/m^2^). Mean daily caloric intake was 2043.7 ± 692.1, of which the mean proportional caloric intake from UPFs was 38.2 ± 16.2%. Intake of UPF was mostly derived from ‘snacks’, ‘beverages’, ‘oils and spreads’ and ‘dairy products’ (Figure S1). High UPF intake (third tertile vs. first tertile) was significantly associated with male gender. Unhealthy lifestyles, including smoking, physical inactivity, and obesity, tended to be higher, and the consumption of a healthy diet tended to be lower among participants consuming high UPF, but these differences were not statistically significant. As expected, participants with high UPF intake had a higher caloric intake, higher proportional carbohydrates and SFA intake, and lower protein intake (Table S2).

### 3.2. The Association between UPF Intake and Colorectal Adenomas

Cases with adenomas were older, had a higher mean BMI, and included a higher proportion of males, smokers, aspirin users, participants with the Mets, and participants undergoing surveillance colonoscopy (Table 1).

Cases and controls did not differ in dietary intake of total calories, calories from different food groups, proportion of calories from macronutrients, and major nutrients, as saturated fatty acids (SFA), fiber, and sodium, but cases had lower intake of mono-unsaturated/SFA ratio. Cases had a higher proportional caloric intake of UPFs, mainly contributed by the following food groups: oils and spreads, dairy products, snacks, and beverages. These differences were also seen for subgroups of cases with advanced adenomas and proximal adenomas (Table 2). 

**Figure 1:**
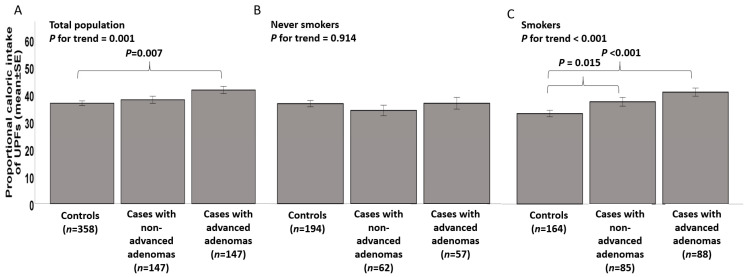
Univariate association between the proportional caloric intake of Ultra-Processed Foods (UPFs) and colorectal adenomas among (**A**) the total population (*n* = 652), (**B**) never smokers (*n* = 315), (**C**) smokers (*n* = 337). Abbreviations: Kcal–Kilocalorie;SE- Standard error

The proportional caloric intake of UPFs was significantly associated with adenoma stage, with a significant positive trend, among the total population and among smokers but not among never-smokers (Figure 1).

**Figure 2:**
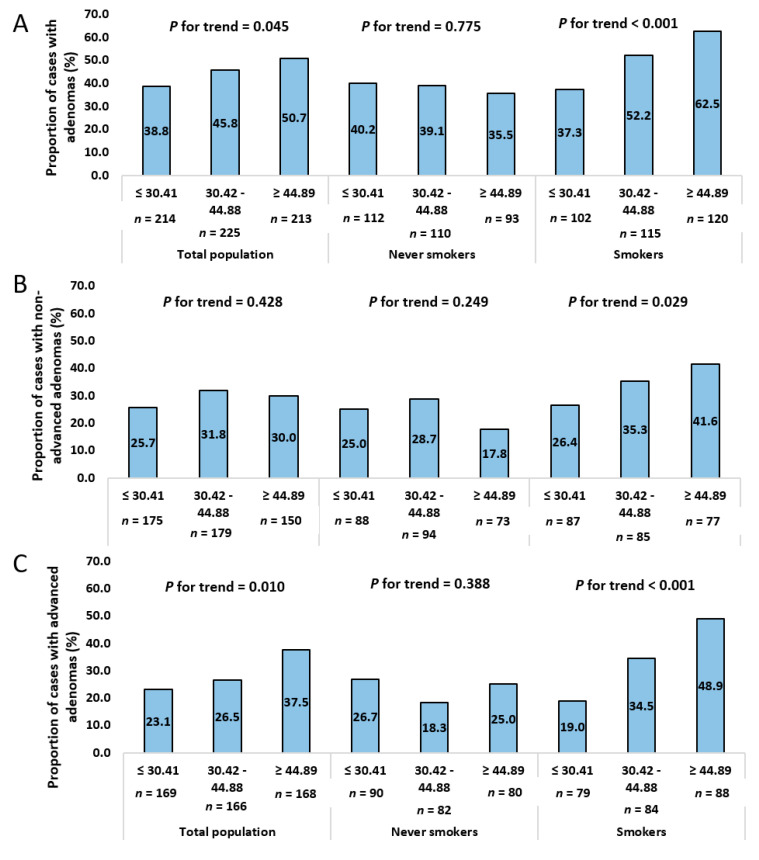
The univariate association between tertiles of proportional caloric intake of Ultra-Processed Foods (UPFs) and (**A**) Adenoma (**B**) Non-advanced adenoma (**C**) Advanced adenoma, stratified by smoking status.

In addition, only among smokers, there was a positive trend in the association between tertiles of proportional caloric intake of UPFs and adenomas, particularly advanced adenomas (Figure 2).

### 3.3. Association between UPF Intake and Colorectal Adenomas and Its Interaction with Smoking

In multivariate analysis, a high UPF intake (third tertile vs. first tertile) was positively associated with adenomas (odds ratio (OR) = 1.75, 95% confidence interval (CI) 1.14–2.68), advanced adenomas (OR = 2.17, 95%CI 1.29–3.65), and proximal adenomas (OR = 2.38, 95%CI 1.37–4.11) among the total study population. A positive dose–response association was detected for adenomas, advanced adenomas, and proximal adenomas. Stratified by smoking status, significant positive dose–response associations between high UPF intake (third tertile vs. first tertile) and colorectal adenomas, non-advanced, advanced, proximal, and distal adenomas were observed only among smokers, and not among never-smokers. There was a significant interaction between smoking and high UPF intake (third tertile vs. first tertile) in relation with adenomas (*p* for interaction = 0.004), non-advanced adenomas (*p* for interaction = 0.019), advanced adenomas (*p* for interaction = 0.007), proximal adenomas (*p* for interaction = 0.026), and distal adenomas (*p* = 0.004) (Table 3).

Among all food groups, high UPF intake (third tertile vs. first tertile) from ‘dairy products’ and ‘snacks’ among smokers and high UPF intake from ‘oils and spreads’ in both smokers and never-smokers were independently associated with colorectal adenomas in multivariate analysis (Table S3).

### 3.4. The Association between UPF Intake and Colonic Adenomas as Compared with Other Major Risk Factors

**Figure 3:**
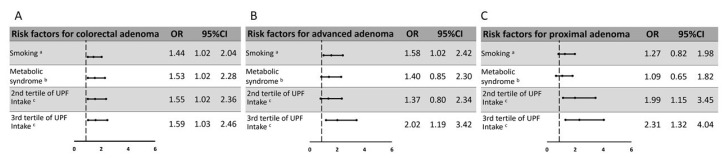
Adjusted association between metabolic and lifestyle-related risk factors and colorectal adenomas. (**A**) All adenoma (**B**) Advanced adenoma (**C**) Proximal adenoma. ^a^ Smoking (ever vs. never).^b^ Metabolic syndrome (yes vs. no). ^c^ UPF intake was defined as tertiles of the proportional caloric intake of UPFs from total caloric intake. The 2nd tertile of UPF (30.4–44.7% of total kcal) and the 3rd tertile (≥44.8% of total kcal) were compared to the 1st tertile (≤30.4% of total kcal). All ORs are adjusted for age, gender, BMI, total kcal, aspirin, indication for colonoscopy, smoking, metabolic syndrome, and high UPF intake. Abbreviations: OR- Odds ratio, CI- Confidence interval, UPF- Ultra-Processed Foods

The association between high intake of UPF and colorectal adenomas, advanced, and proximal adenomas was as strong as that of established risk factors for colorectal cancer, such as smoking and the Mets, in a multivariate model with all risk factors adjusted for confounding factors and for one another (Figure 3).

## 4. Discussion

Processed food, a key component of the western diet, is prevalent among various food groups [13], and has been shown to contribute up to 60% of the daily kcal intake of adults [26], 40% of children [27], and 20% of infants [28]. UPF intake has been studied in association with various health outcomes, including cancer, and specifically, CRC [16], but evidence regarding its association with pre-malignant colorectal polyps is lacking. Our study findings show a positive independent association between UPF intake and colorectal adenomas.

We observed strong positive dose–response associations between high UPF intake and colorectal adenomas of both types and of both colorectal locations. These associations were observed only among smokers, and a significant interaction was detected between smoking and high UPF intake in relation to all colorectal adenoma categories. The association between high UPF intake and colorectal adenomas was as strong as the association between smoking or the Mets and adenomas, well-established risk factors for colorectal carcinogenesis, which have been previously suggested as CRC screening referral factors [29].

As expected, participants who consumed higher proportions of UPF also consumed significantly more calories, SFA, and carbohydrates. Western lifestyle characteristics, such as smoking, physical inactivity, obesity, and the consumption of a healthy diet, were different between participants characterized by different intake levels of UPF, but these differences were not significant. Cases and controls differed in UPF intake but not in intake of other dietary components of the western diet, such as fiber, sodium, and fat. Additionally, the associations between UPF intake and adenomas were consistent across types and locations of adenomas, independent of total caloric intake and BMI, which were adjusted for in multivariate analysis. Therefore, it is reasonable to assume that the association of UPF with adenomas is not just a reflection of an association with an unhealthy western diet. These results strengthen the independent association between UPF intake and colorectal neoplasia.

This association is supported by biological mechanisms linking CRC development and growth, with various synthetic components of processed food. These include nitrates and nitrites [30] in processed meats, and other less studied food additives, such as monosodium glutamate, titanium dioxide [31], high-fructose corn syrup [32], and synthetic dyes [33]. Indeed, the major sources of UPFs that were found to be related with colorectal adenomas were ‘snacks’, ‘oils and spreads’, and ‘dairy products’; all contain significant amounts of the above-mentioned components and food additives. Surprisingly, the ‘dairy products’ food group, which is considered an important part of the Dietary Approach to Stop Hypertension (DASH) diet and the Mediterranean diet [34], included 40% of the kcal from ultra-processed dairy products. Cases with colorectal adenomas consumed higher proportions of ultra-processed dairy products, and dairy products were positively associated with colorectal adenomas among smokers in multivariate analysis. Previous reports have shown dairy products to be protective of colorectal neoplasia, but results have been inconclusive [34,35]. Given that none of these previous reports had differentiated between processed and non-processed dairy products, these inconsistencies may be attributed to this potentially important distinction. Among our study population, the intake of ultra-processed ‘meat, poultry, and fish’ was about 10% of the total kcal, nearly a third of the intake reported in other cohorts of the western world [36]. This may explain the finding that high intake of processed ‘meat, poultry, and fish’ was not significantly associated with colorectal adenomas in this cohort, unlike the findings of others [37]. Another explanation may be that the intake of UPF as a whole is the exposure of importance, regardless of the food group the UPF is oriented from.

The positive association between UPF intake and adenomas was stronger with advanced adenomas, and may reflect a potential role of UPFs in colorectal neoplasia progression. The association with proximal adenomas differs from previous reports that link diet and smoking with distal adenomas [7,8]. Interestingly, the associations persisted following adjustments for various potential confounding factors among smokers but not among non-smokers, yielding a significant interaction. Importantly, in our study, smoking and other unhealthy lifestyle characteristics were not associated with UPF intake, therefore potential confounding by these factors is unlikely. Additionally, a dose-dependent association was detected between UPF intake and colorectal adenomas, with a significant positive trend seen only among smokers. Previous studies have demonstrated interactions between diet and smoking in their association with colorectal neoplasia, but these have mainly been attributed to meat intake [38,39] and plant-based diets [40]. To our knowledge, none of these studies have addressed UPFs. Therefore, we believe that this is the first report of an association between UPF intake and colorectal adenomas, and of its interaction with smoking. One potential mechanism to explain this interaction is the joint effects of smoking and diet on the gut microbiome [41], which can in turn influence CRC risk. The main proposed mechanisms linking the microbiome with colorectal neoplasia include secretion of oncogenic microbial metabolites, such as secondary bile acids, and activation and promotion of an inflammatory response in the gut mucosa, which further promote the growth of tumor cells, and inhibit apoptosis [42,43,44]

The limitations of this study include the lack of temporal sequence, which does not permit a causal inference. Cases and controls were recruited from the same population and had comparable socioeconomic characteristics, thus minimizing a potential selection bias. In terms of external validity, this study population was intentionally selected to represent a population with low to medium risk for colorectal polyp; thus, our findings cannot be generalized to high-risk populations. We attempted to minimize a potential confounding effect and elaborate on effect modification by stratification across smoking status, which is strongly associated with CRC, and adjustment in multivariate analysis. Still, residual confounding may still exist. Nutritional data were collected within a single country, which may impact on the generalizability across populations with different diets. Information bias, and particularly recall bias, on lifestyle characteristics may exist, due to the retrospective nature of the study. This was minimized by a uniform structured lifestyle and dietary questionnaire, which was assessed in the same manner among cases and controls, all blinded to the study hypothesis to prevent differential bias.

## 5. Conclusions

Among smokers, high UPF intake is strongly and independently associated with colorectal adenomas, especially for advanced and proximal adenoma. The results highlight the need to further investigate the role of UPF as an independent risk factor for colorectal neoplasia, and its interaction with smoking, in larger prospective studies, incorporating microbiome analysis. As UPF intake increases worldwide, especially among children and young adults, its negative implications should be targeted for CRC prevention.

## Figures and Tables

**Table 1:** Characteristics of the study population, and comparison between cases with adenomas and controls.

	Controls (*n* = 358)	Cases with Adenoma (*n* = 294)	*p*	Cases with Non-Advanced Adenoma (*n* = 147)	*p*	Cases with Advanced Adenoma (*n* = 147)	*p*	Cases with Proximal Adenoma (*n* = 143)	*p*	Cases with Distal Adenoma (*n* = 151)	*p*
Age (years)	57.9 ± 6.8	59.4 ± 9.6	0.004	59.3 ± 6.5	0.037	59.7 ± 6.2	0.006	60.5 ± 5.8	<0.001	58.5 ± 6.7	0.337
Gender (% male)	46.2	56.3	0.012	54.5	0.100	57.6	0.023	49.3	0.558	62.4	0.001
Low socio-economic status ^a^ (%)	5.6	8.1	0.220	8.5	0.245	7.7	0.384	8.0	0.332	8.1	0.306
Never smoked (%)	54.2	41.2	0.002	42.2	0.077	40.4	0.002	44.8	0.052	38.2	0.003
Past smoker (%)	33.2	37.8		40.1		35.6		35.0		40.1	
Current smoker (%)	12.6	21.1		17.7		24.0		20.3		20.8	
BMI (kg/m^2^)	27.3 ± 4.8	29.0 ± 5.8	<0.001	29.0 ± 5.0	0.001	28.9 ± 6.5	0.010	28.3 ± 4.9	0.043	29.5 ± 6.5	<0.001
Aspirin use (%)	23.5	32.6	0.011	35.0	0.010	31.3	0.079	31.4	0.075	33.6	0.022
Physical inactivity ^b^ (%)	40.5	47.2	0.094	46.1	0.245	47.9	0.127	51.1	0.031	43.8	0.474
Metabolic syndrome (%)	42.9	66.1	<0.001	66.9	<0.001	64.3	<0.001	65.4	<0.001	66.2	<0.001
Indication for colonoscopy											
Screening (%)	60.5	39.9	<0.001	39.9	<0.001	39.6	<0.001	40.7	<0.001	38.9	<0.001
Alarming symptoms (%)	34.0	35.4		30.8		40.3		30.7		39.6	
Surveillance (%)	5.5	24.7		29.4		20.1		28.6		21.5	

^a^ Low socio-economic status–A combination of low education (<12 years of school education) and low income (< the group 1st Q of monthly household income) ^b^ Physical inactivity–Reported no intentional exercise or less than 20 min/week of exercise, which leads to increased heart rate and/or sweating. Abbreviations: BMI-Body Mass Index

**Table 2:** Comparison of dietary intake and Ultra-Processed Food (UPF) intake between cases and controls.

	Controls (*n* = 358)	Cases with Adenoma (*n* = 294)	*p*	Cases with Non-Advanced Adenoma (*n* = 147)	*p*	Cases with Advanced Adenoma (*n* = 147)	*p*	Cases with Proximal Adenoma (*n* = 143)	*p*	Cases with Distal Adenoma (*n* = 151)	*p*
Dietary intake											
Caloric intake (Kcal/day)	2031 ± 687	2027 ± 705	0.944	2058 ± 683	0.687	1994 ± 723	0.596	2020 ± 651	0.874	2036 ± 753	0.932
Protein (% of total kcal)	18.2 ± 4.4	18.3 ± 4.6	0.753	18.5 ± 4.5	0.527	18.1 ± 4.8	0.838	17.3 ± 4.2	0.023	19.4 ± 4.8	0.012
Fat (% of total kcal)	36.4 ± 6.6	35.8 ± 6.4	0.215	35.8 ± 6.6	0.399	36.0 ± 6.3	0.498	36.0 ± 6.2	0.563	35.7 ± 6.7	0.274
SFA (% of total kcal)	12.3 ± 3.7	12.2 ± 3.7	0.795	12.0 ± 3.4	0.381	12.5 ± 4.1	0.473	12.2 ± 3.4	0.754	12.3 ± 4.0	0.868
MUFA/SFA ratio	1.06 ± 0.45	1.00 ± 0.33	0.041	1.03 ± 0.36	0.322	0.99 ± 0.32	0.037	1.02 ± 0.34	0.233	0.99 ± 0.33	0.041
Carbohydrates (% of total kcal)	41.6 ± 8.7	42.2 ± 8.6	0.354	42.0 ± 8.1	0.702	42.3 ± 9.0	0.432	43.4 ± 8.2	0.046	41.1 ± 8.9	0.536
Fiber (gr/day)	23.6 ± 11.5	27.4 ± 12.8	0.547	24.5 ± 10.7	0.417	21.5 ± 12.8	0.076	23.1 ± 12.0	0.672	23.0 ± 12.0	0.611
Sodium (mg/day)	2774 ± 1048	2773 ± 1037	0.975	2876 ± 1056	0.321	2669 ± 1011	0.299	2665 ± 1014	0.284	2876 ± 1048	0.313
Total caloric intake from food groups											
Bread, pastries and starch (kcal)	403.1 ± 230.4	420.2 ± 239.6	0.360	450.8 ± 251.7	0.044	391.9 ± 223.9	0.621	414.7 ± 237.3	0.622	425.1 ± 251.74	0.340
Snacks (kcal)	208.5 ± 218.7	207.8 ± 202.3	0.966	182.4 ± 175.9	0.206	233.8 ± 224.4	0.249	233.1 ± 201.5	0.252	184.2 ± 200.3	0.246
Beverages (kcal)	150.2 ± 175.1	174.0 ± 206.8	0.118	161.3 ± 211.0	0.567	181.3 ± 195.8	0.089	180.3 ± 217.1	0.117	166.9 ± 196.9	0.363
Oils and spreads (kcal)	188.1 ± 143.9	184.5 ± 176.8	0.779	203.3 ± 192.5	0.329	167.9 ± 160.3	0.179	194.5 ± 205.2	0.686	175.7 ± 144.7	0.392
Dairy (kcal)	245.0 ± 200.4	242.0 ± 203.2	0.894	237.9 ± 177.5	0.760	250.8 ± 228.8	0.736	231.0 ± 184.0	0.514	256.6 ± 221.5	0.528
Meat, poultry and fish (kcal)	288.8 ± 191.9	304.0 ± 231.0	0.368	314.7 ± 257.3	0.224	292.1 ± 203.2	0.869	258.3 ± 169.3	0.102	346.8 ± 269.6	0.007
The proportional caloric intake of UPFs by food group											
Total UPF kcal/total kcal (%)	36.9 ± 16.4	39.2 ± 16.4	0.043	38.2 ± 15.6	0.251	40.3 ± 16.9	0.019	40.4 ± 16.2	0.016	38.0 ± 16.4	0.422
Bread, pastries and starch UPF kcal/group kcal (%)	19.2 ± 24.3	17.4 ± 22.2	0.327	19.0 ± 22.8	0.936	15.7 ± 22.2	0.129	18.3 ± 22.0	0.686	16.4 ± 22.4	0.229
Snacks UPF kcal/group kcal (%)	71.1 ± 31.2	77.3 ± 27.5	0.010	76.2 ± 28.5	0.102	78.3 ± 26.4	0.020	76.5 ± 27.8	0.084	77.9 ± 7.4	0.030
Beverages UPF kcal/group kcal (%)	59.8 ± 37.9	66.8 ± 37.9	0.026	64.7 ± 39.0	0.210	69.1 ± 36.9	0.016	68.6 ± 36.2	0.022	64.9 ± 37.7	0.187
Oils and spreads UPF kcal/group kcal (%)	56.2 ± 33.3	66.1 ± 32.5	<0.001	66.9 ± 33.4	0.001	64.9 ± 32.2	0.009	64.1 ± 32.9	0.018	67.8 ± 32.2	<0.001
Dairy UPF kcal/group kcal (%)	35.5 ± 31.2	43.5 ± 32.6	0.002	42.5 ± 31.1	0.025	44.9 ± 34.1	0.005	43.5 ± 30.9	0.011	43.5 ± 34.1	0.021
Meat, poultry and fish UPF kcal/group kcal (%)	8.8 ± 14.2	8.8 ± 13.0	0.327	9.7 ± 12.9	0.283	7.7 ± 12.9	0.399	18.3 ± 22.0	0.883	9.5 ± 14.2	0.370

Abbreviations: Kcal–Kilocalorie, MUFA-Mono-Unsaturated Fatty Acid, SFA-Saturated Fatty Acids, UPFs-Ultra-Processed Foods.

**Table 3:** The adjusted association between high Ultra-Processed Foods (UPFs)intake, and colorectal adenomas as compared to controls, stratified by smoking status.

		Cases with Adenoma OR (95%CI) *p*	Cases with Non-Advanced Adenoma OR (95%CI) *p*	Cases with Advanced Adenoma OR (95%CI) *p*	Cases with Proximal Adenoma OR (95%CI) *p*	Cases with Distal Adenoma OR (95%CI) *p*
Total study population (*n* = 652) Cases/controls 294/358	1st tertile of UPF intake					
	Cases/controls	83/131	44/131	39/131	36/131	47/131
		Ref.	Ref.	Ref.	Ref.	Ref.
	2nd tertile of UPF intake ^b^					
	Cases/controls	103/122	58/122	45/122	52/122	51/122
		1.58 (1.04–2.40) 0.030	1.67 (1.00–2.78) 0.048	1.39 (0.81–2.38) 0.219	1.99 (1.15–3.44) 0.013	1.32 (0.79–2.19) 0.278
	3rd tertile of UPF intake ^b^					
	Cases/controls	108/105	45/105	63/105	55/105	53/105
		1.75 (1.14–2.68) 0.009	1.31 (0.76–2.25) 0.325	2.17 (1.29–3.65) 0.003	2.38 (1.37–4.11) 0.002	1.39 (0.82–2.34) 0.212
Never smokers (*n* = 315) Cases/controls 121/194	1st tertile of UPF intake					
	Cases/controls	45/67	22/67	23/67	22/67	23/67
		Ref.	Ref.	Ref.	Ref.	Ref.
	2nd tertile of UPF intake ^b^					
	Cases/controls	43/67	28/67	15/67	25/67	18/67
		1.10 (0.62–1.97) 0.730	1.44 (0.71–2.95) 0.309	0.67 (0.31–1.47) 0.326	1.32 (0.63–2.74) 0.453	0.88 (0.41–1.87) 0.748
	3rd tertile of UPF intake ^b^					
	Cases/controls	33/60	13/60	20/60	17/60	16/60
		0.84 (0.45–1.55) 0.589	0.56 (0.24–1.29) 0.176	1.02 (0.48–2.15) 0.947	0.90 (0.41–1.98) 0.808	0.72 (0.33–1.60) 0.432
Smokers ^a^ (*n* = 337) Cases/controls 173/164	1st tertile of UPF intake					
	Cases/controls	38/64	22/64	16/64	14/64	24/64
		Ref.	Ref.	Ref.	Ref.	Ref.
	2nd tertile of UPF intake ^b^					
	Cases/controls	60/55	30/55	30/55	27/55	33/55
		2.43 (1.31–4.52) 0.005	2.06 (0.96–4.39) 0.060	2.86 (1.30–6.26) 0.009	3.40 (1.43–8.05) 0.005	1.97 (0.96–4.02) 0.061
	3rd tertile of UPF intake ^b^					
	Cases/controls	75/45	32/45	43/45	38/45	37/45
		3.54 (1.90–6.61) < 0.001	2.60 (1.20–5.63) 0.015	4.76 (2.20–10.30) < 0.001	6.23 (2.67–14.52) < 0.001	2.49 (1.21–5.13) 0.013
P for interactionbetween UPF intake and smoking status ^c^	2nd tertile of UPF intake	0.100	0.533	0.017	0.137	0.159
	3rd tertile of UPF intake	0.004	0.019	0.007	0.026	0.004

ORs are adjusted for age, gender, BMI, total kcal, aspirin use and indication for colonoscopy. ^a^ Smoking is defined as ever (past/present) smoking. ^b^ Ultra-Processed Foods (UPFs) intake was defined as the proportional caloric intake of UPFs from total caloric intake. The 2nd tertile of UPF (30.4–44.7% of total kcal) and the 3rd tertile (≥44.8% of total kcal) were compared to the 1st tertile (≤30.4% of total kcal). ^c^ The interaction between UPF intake and smoking status, adjusted for all parameters of the model, smoking and high UPF intake. Abbreviations: OR- Odds ratio, CI- Confidence interval, UPF- Ultra-Processed Food

## Supplementary Materials

The following are available online at https://www.mdpi.com/2072-6643/12/11/3507/s1, Table S1. Un-processed and Processed vs. Ultra-processed Food groups, Figure S1. The proportional caloric intake of UPFs in the total diet and within food groups; Table S2. The association between high UPF intake (≥44.8% of total kcal) and lifestyle characteristics; Table S3. The adjusted association between high UPF intake from food groups, and colorectal adenoma as compared to controls, stratified by smoking status.
