# Supplementary material for: Ultra-Processed Food Intake and Smoking Interact in Relation with Colorectal Adenomas
Source: Nutrients. 2020 Nov 14;12(11):3507. doi: 10.3390/nu12113507 (PMC7698317; doi:10.3390/nu12113507)
Supplement: Supplementary file 1 [file nutrients-12-03507-s001.pdf]

Supplementary Materials: The following are available online at www.mdpi.com/xxx/s1, **Table A1.** Un-processed and Processed vs. Ultra-processed Food groups, Figure S1. The proportional caloric intake of UPFs in the total diet and within food groups; **Table A2**. The association between high UPF intake (≥44.8% of total kcal) and lifestyle characteristics; **Table A3.** The adjusted association between high UPF intake from food groups, and colorectal adenoma as compared to controls, stratified by smoking status

Table 1. Un-processed and Processed vs. Ultra-processed Food groups.

**Unprocessed and processed**	**Ultra-processed**
Bread, pastries and starch	
White bread<br>Black bread<br>Whole wheat bread<br>Pitta bread<br>Rice pasta<br>Semolina<br>Baked or boiled potato	Diet bread with fiber<br>Diet white bread<br>Crackers<br>Cornflakes<br>Pizza<br>Burekas<br>Malawach
Snacks	
Home-made cake<br>Jam<br>Dried fruit	Chocolate roll cake<br>Busquets and bought cookies<br>Waffle<br>Cookies<br>Milk chocolate<br>Chocolate bar<br>Chocolate spread<br>Cream ice cream<br>Non-dairy ice cream<br>Energy bar<br>Diet energy bar<br>Salty snacks<br>Potato chips
Beverages	
Fresh fruit juice<br>Fresh carrot juice<br>Soda water<br>Green tea<br>Black tea<br>Water	Sugar sweetened beverages<br>Diet sugar sweetened beverages<br>Processed fruit juice<br>Root beer<br>Iced tea<br>Flavored water<br>Iced coffee<br>Diet iced tea
Oils and spreads	
Olives<br>Olive oil<br>Canola oil<br>Soy oil<br>Corn oil<br>Sunflower oil<br>Nuts	Margarine<br>Bought Humus paste<br>Bought tachina paste<br>Bought cabbage carrot mayonnaise salad
Dairy	
Milk 1-3% fat<br>Yogurt unsweetened<br>Cream cheese<br>Sour cream<br>Butter	Milk beverages<br>Sweetened yogurt and dairy products<br>Diet sweetened yogurt<br>Chadder and yellow cheese
Meat poultry fish	
Beef steak, roast, ground meat products<br>Internal organs of beef and chicken	Processed fried chicken<br>Beef hamburger

Unprocessed and processed	Ultra-processed
Chicken breast or leg cooked or fried	Sausage, cold cuts, pastrami, salami
Lamb	Preserved meat
Pork	Meat substitutes
Duck	
Fresh fish	
Salted fish	
Canned tuna or sardines	

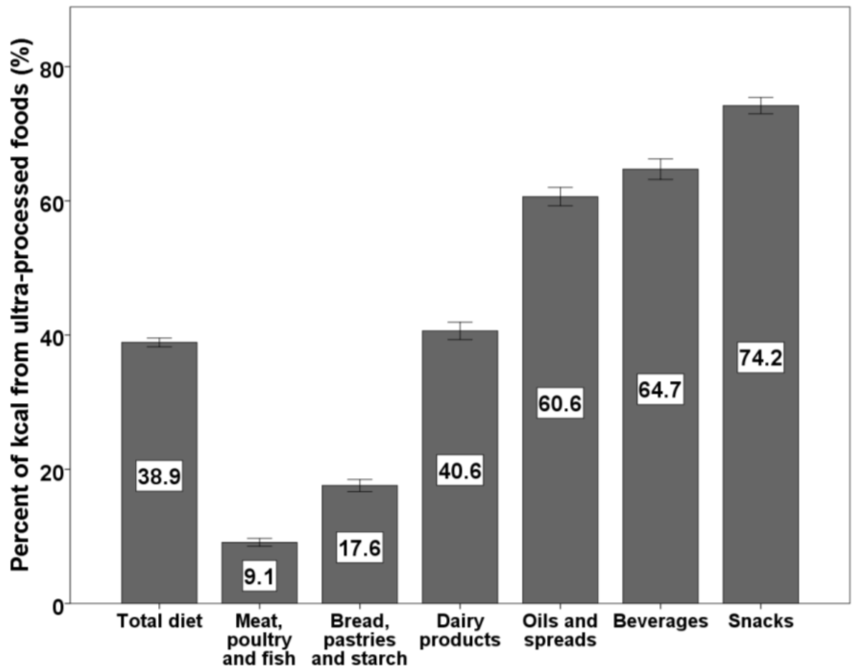

Figure 1. The proportional caloric intake of UPFs in the total diet and within food groups within the total study population.

Table 2. The association between high UPF intake (≥44.8% of total kcal) and lifestyle characteristics.

	UPF intake ≤30.41% of total kcal (n=214)	UPF intake 30.42-44.88 of total kcal (n=225)	UPF intake ≥44.89% of total kcal (n=213)	P
Age (years) (mean±std)	58.8±6.8	57.8±6.4	58.9±6.7	0.167
**Gender (% male)**	**49.4**	**45.2**	**57.7**	**0.023**
Smoking [a] (%)	50.2	51.3	58.1	0.180
Obesity [b] (%)	27.3	27.9	33.9	0.228
Physical inactivity [c] (%)	43.4	44.5	44.6	0.958
Healthy diet index [d] (%)	53.7	45.2	46.2	0.136
**Caloric intake (Kcal/day)**	**1883±628**	**2032±680**	**2218±735**	**<0.001**
**Protein (% of total kcal)**	**20.0±4.6**	**18.2±3.9**	**16.3±4.1**	**<0.001**
Fat (% of total kcal)	36.6±6.6	35.8±6.0	36.3±7.0	0.427
**SFA (% of total kcal)**	**12.1±3.5**	**12.7±4.1**	**12.3±3.6**	**0.046**
**Carbohydrates (% of total kcal)**	**39.4±8.4**	**42.3±7.8**	**44.1±9.2**	**<0.001**
Fiber (gr/day)	22.4±10.6	24.2±13.0	23.4±11.9	0.260
Sodium (mg/day)	2745±1027	2750±979	2842±1053	0.516
Aspirin use (%)	23.8	27.0	31.2	0.200

[a] Smoking is defined as ever (past/present) smoking; [b] Obesity is defined as BMI>30 kg/m^2; [C] Physical inactivity – Reported no intentional exercise or less than 20 min/ week of exercise which leads to increased heart rate and/or sweating; [d] A healthy dietary index was defined if participants reported consuming at least five of the American Heart Association (AHA) healthy diet components.

Table 3. The adjusted association between high UPF intake from food groups, and colorectal adenoma as compared to controls, stratified by smoking status.

	**Total study population**		**Never smokers**		**Smokers**	
	**Cases (n=294)/ Controls (n=358)**	**OR (95%CI) P**	**Cases (n=121)/ Controls (n=194)**	**OR (95%CI) P**	**Cases (n=173)/ Controls (n=164)**	**OR (95%CI) P**
**Bread, pastries and starch**						
1st tertile (≤2.03% UPF kcal/group kcal)	98/117	Ref.	36/59	Ref.	62/58	Ref.
2nd tertile (2.04-19.73% UPF kcal/group kcal)	102/121	1.00 (0.66-1.52) 0.976	47/72	1.21 (0.66-2.21) 0.522	55/49	0.85 (0.46-1.55) 0.604
3rd tertile (≥19.74% UPF kcal/group kcal)	94/120	1.05 (0.69-1.60) 0.799	38/63	1.13 (0.61-2.11) 0.687	56/57	0.99 (0.55-1.78) 0.978
**Snacks**						
1st tertile (≤71.37% UPF kcal/group kcal)	86/120	Ref.	38/60	Ref.	48/60	Ref.
2nd tertile (71.38-94.90% UPF kcal/group kcal)	98/142	0.99 (0.65-1.52) 0.998	42/85	0.86 (0.47-1.55) 0.626	56/57	1.19 (0.64-2.23) 0.575
3rd tertile (≥94.91% UPF kcal/group kcal)	110/96	**1.63 (1.06-2.49) 0.023**	41/49	1.29 (0.68-2.42) 0.425	69/47	**2.08 (1.15-3.77) 0.015**
**Beverages**						
1st tertile	84/131	Ref.	35/77	Ref.	49/54	Ref.

	Total study population		Never smokers		Smokers	
	Cases (n=294)/ Controls (n=358)	OR (95%CI) P	Cases (n=121)/ Controls (n=194)	OR (95%CI) P	Cases (n=173)/ Controls (n=164)	OR (95%CI) P
(≤50.0% UPF kcal/group kcal)						
2nd tertile (50.01-95.83% UPF kcal/group kcal)	111/124	1.26 (0.82-1.94) 0.274	48/69	1.26 (0.68-2.31) 0.454	63/55	1.33 (0.71-2.52) 0.366
3rd tertile (≥95.84% UPF kcal/group kcal)	99/103	1.34 (0.88-2.05) 0.164	38/48	1.54 (0.82-2.89) 0.172	61/55	1.14 (0.64-2.05) 0.638
**Oils and spreads**						
1st tertile (≤49.51% UPF kcal/group kcal)	81/142	Ref.	36/82	Ref.	45/60	Ref.
2nd tertile (49.52-85.22% UPF kcal/group kcal)	104/126	1.40 (0.92-2.13) 0.108	38/66	1.25 (0.68-2.30) 0.468	66/60	1.52 (0.84-2.74) 0.161
3rd tertile (≥85.23% UPF kcal/group kcal)	109/90	**2.20 (1.42-3.40) <0.001**	47/46	**2.49 (1.33-4.64) 0.004**	62/44	**2.99 (1.07-3.71) 0.029**
**Dairy**						
1st tertile (≤17.94% UPF kcal/group kcal)	79/133	Ref.	35/66	Ref.	44/67	Ref.
2nd tertile (17.95-53.23% UPF kcal/group kcal)	107/127	**1.61 (1.06-2.46) 0.025**	47/75	1.48 (0.81-2.70) 0.195	60/52	1.79 (0.98-3.27) 0.056
3rd tertile (≥53.24% UPF kcal/group kcal)	108/98	**1.92 (1.26-2.93) 0.002**	39/53	1.47 (0.79-2.75) 0.219	69/45	**2.44 (1.36-4.36) 0.003**
**Meat, poultry and fish**						
1st tertile (≤0.78% UPF kcal/group kcal)	95/137	Ref.	47/75	Ref.	48/62	Ref.
2nd tertile (0.79-8.45% UPF kcal/group kcal)	102/117	1.19 (0.79-1.79) 0.390	38/63	0.90 (0.50-1.61) 0.726	64/54	1.53 (0.86-2.73) 0.147
3rd tertile (≥8.46% UPF kcal/group kcal)	97/104	1.23 (0.80-1.87) 0.332	36/56	1.05 (0.57-1.95) 0.864	61/48	1.39 (0.77-2.52) 0.270

UPF intake was categorized as tertiles of the proportional caloric intake of UPFs from total caloric intake within each food group. The 3rd and 2nd tertiles were compared to the 1st tertile. ORs are adjusted **for** age, gender, BMI, total kcal, aspirin, use, and indication for colonoscopy.
